# Assessment of Pediatric Kidney Transplant Experience and Exposure During Pediatric Nephrology Fellowship Training

**DOI:** 10.34067/KID.0000000000000177

**Published:** 2023-06-08

**Authors:** Elizabeth Spiwak, Sharon M. Bartosh, Jodi Smith, Michael E. Seifert, Sandra Amaral, Roshan P. George

**Affiliations:** 1 Department of Pediatric Nephrology, Peyton Manning Children's Hospital, Indianapolis, Indiana; 2 Department of Pediatrics, University of Wisconsin Medical School, Madison, Wisconsin; 3 Department of Pediatrics, University of Washington School of Medicine, Seattle, Washington; 4 Division of Pediatric Nephrology, University of Alabama at Birmingham, Birmingham, Alabama; 5 Departments of Pediatrics and Biostatistics, Epidemiology, and Informatics, Children's Hospital of Philadelphia and University of Pennsylvania, Philadelphia, Pennsylvania; 6 Division of Pediatric Nephrology, Emory University, Atlanta, Georgia

**Keywords:** children, kidney transplantation, pediatric kidney transplantation, pediatric nephrology

## Abstract

- There have been no published studies evaluating competency or perceived competency in kidney transplant–related knowledge and skills among graduates of pediatric nephrology fellowships.
- There are also no uniform specific transplant requirements within the pediatric nephrology fellowship.
- We aim to further understand this potential gap to rectify it in the future.

## Background

In 2017, the Organ Procurement and Transplant Network and the United Network for Organ Sharing (UNOS) updated the requirements to serve as the primary transplant physician for Pediatric Kidney Transplant Programs. One of the pathways to fulfill the requirements relies on experience gained during completion of a pediatric nephrology fellowship (Table 1).^1^ Pediatric nephrology fellowship programs are 3 years in length, with a minimum of 12 months dedicated to research and scholarly activities (33%).^2^ According to the Accreditation Council for Graduate Medical Education, there are 48 accredited programs for Pediatric Nephrology Fellowship for the academic year 2022–2023.^3^

**Table 1.** Requirements for primary kidney transplant physician for a 3-year pediatric nephrology fellowship pathway

1. Direct involvement in primary care of ten or more transplant kidney recipients for at least 6 months from the time of transplant
2. Experience occurred with a qualified kidney transplant physician and surgeon at a kidney transplant program that performs an average of at least ten pediatric kidney transplants per year
3. Direct involvement in evaluation of 25 potential kidney recipients, including participation in selection committee meetings
4. Maintain current working knowledge of kidney transplantation, defined as direct involvement in kidney transplant patient care over the past 2 years
5. Observation of at least three kidney procurements, including at least one deceased donor and one living donor
6. Observation of at least three kidney transplants involving a pediatric recipient

Adult nephrology 2-year fellowships offer a third dedicated additional year of postgraduate training for those interested in achieving expertise in kidney transplant. No such additional transplant-specific training opportunities are available for pediatric nephrology fellows who already train for 3 years. In 2009, a self-perceived competency evaluation demonstrated that recent adult fellowship trainees felt well-trained and competent in caring for kidney transplant patients, including the performance of kidney transplant biopsies.^4^ There is no similar published literature of recent graduates of pediatric nephrology fellowships. Given the lack of a core transplantation curriculum in fellowship training and lower kidney transplant volumes in children, it is unclear how many pediatric nephrology fellows perceive that they obtain the minimum experience required to qualify as a primary pediatric kidney transplant physician. We conducted a survey of pediatric nephrology fellows to examine current transplant-specific training.

## Methods

A Research Electronic Data Capture survey was created by the Transplant Interest Group of the American Society of Pediatric Nephrology (ASPN). The survey was distributed through the ASPN Fellowship Training Program Directors Committee to optimize responses from current pediatric nephrology fellows or recent graduates. The survey included 14 questions relating to the participants' exposure to pretransplant and post-transplant management and procedures during training. Questions were primarily on the Likert scale, with two questions allowing for explanation and an open-ended call for comments. The training institution was not identified to promote anonymity. All survey responses were included for review. Survey questions are included in Supplemental Appendix 1.

## Results

**Figure 1.**
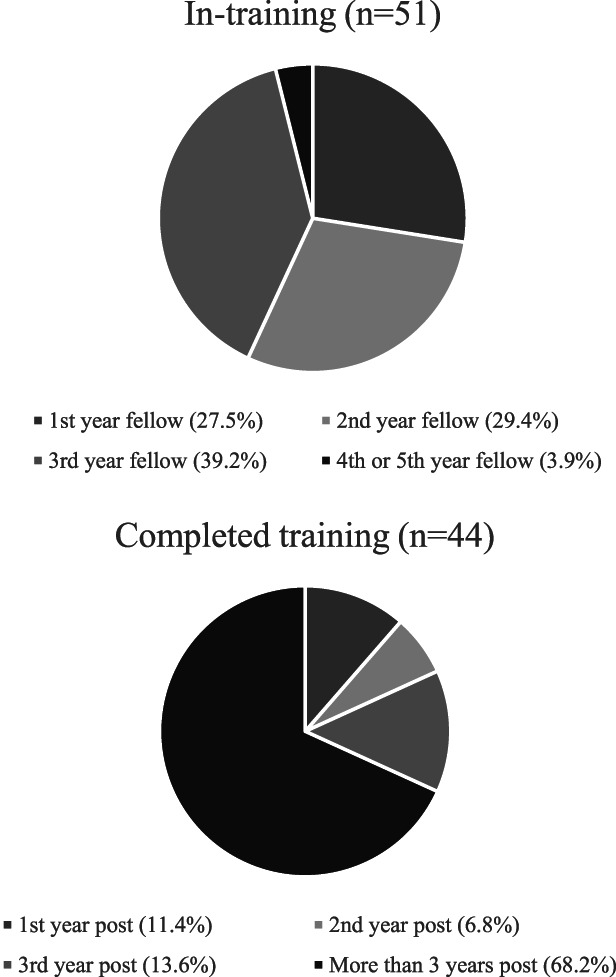
Demographics of survey participants.

A total of 95 participants responded, with 51 (54%) still in training. The remainder participants were recent graduates, with 68% being ≥3 years postfellowship (Figure 1).

### Transplant Candidate Evaluations

Just over a third of respondents (37%) reported that they were involved in 1–5 kidney transplant evaluations during fellowship, and 12% reported involvement in more than 20 evaluations. No direct involvement with evaluations of kidney transplant candidates was reported by 19% respondents.

### Kidney Procurements and Transplant Surgeries

Most respondents observed no kidney procurements during their fellowship training (53% no living donors and 77% no deceased donors). Of those who did observe living donor procurements, 41% observed 1–3, with 6% observing >4. Among those who observed deceased donor procurements, 22% observed 1–3 and 1% observed 4–6. Regarding organ placement, 41% of the respondents did not observe any living donor kidney transplants during their fellowship training. Fifty-one percent of respondents observed 1–3 living donor kidney transplants, with 6% observing 4–6 and 2% observing >12.

### Post-Transplant Care

Survey participants reported that 46% had managed recent pediatric kidney transplant patients directly caring for more than 12 recipients, 25% caring for 10–12, and 25% caring for <10. Only 3% of respondents reported caring for no recent pediatric kidney transplants during training. In assessing longitudinal care (up to 6 months) of recent pediatric kidney recipients, 32% of responders cared for more than 12 patients, 18% cared for 7–12, 18% cared for 4–6, and 19% cared for 1–3. No experience at all in longitudinal care of recent pediatric kidney transplants was reported by 14% of the respondents. Long-term care (more than 6 months) of pediatric kidney transplant recipients was reported as follows: 36% followed 1–10 recipients long term, 30% followed 11–30 long term, and 20% followed more than 30 long term. No long-term care of a pediatric kidney transplant recipient was reported by 15% of respondents.

### Kidney Allograft Biopsies

Kidney transplant biopsies were performed in a variety of center-specific scenarios, including with assistance from an ultrasound technician (59%), interventional radiologist (19%), the attending nephrologist alone (4%), or other (18%). These scenarios were often directed by the anatomic location of the transplanted kidney. A few respondents noted that transplant surgeons perform kidney transplant biopsies, with no specification about whether nephrology trainees were involved. The highest proportion of respondents (37%) had performed >20 kidney transplant biopsies, followed by 32% performing between 1 and 10, 21% performing between 11 and 20 and 11% performing no transplant biopsies.

### Advocacy

Most participants (86%) had not attended any UNOS regional or committee meetings during fellowship training.

### Center-Specific Requirements

Most responders (81%) reported that their fellowship program had no specific requirements for the expected number of kidney transplant biopsies, procurements, surgeries, and post-transplant care encounters. Those who trained at programs with specific requirements reported that 10–30 biopsies were required (no specific number of transplant biopsies), along with 1–3 procurements and 1–3 transplant surgeries.

## Discussion

We observed a high degree of variability in exposure to transplantation among trainees and recent graduates of pediatric nephrology fellowship programs. The finding that only half of the trainees observed kidney transplantation surgery from a living donor and less than half observed deceased or living donor procurement is of particular concern. Pediatric nephrology fellowship curriculums do not consistently contain transplant-specific content. In adult nephrology, the 1-year fellowship opportunity for those interested in achieving expertise in transplant nephrology seems to produce greater confidence and perceived self-competency in their ability to care for adult kidney transplant patients. Pediatric nephrology fellows, who are already doing a 3-year fellowship, are expected to receive training for the care of pediatric kidney transplant recipients during that time frame. A similar study was completed in pediatric transplant hepatology fellowship programs and also found wide variability in trainee experiences.^5^ In addition to procedural experiences, they also evaluated the formal lecture experience and have created a new virtual lecture experience to ensure consistency among training programs. There are also Accreditation Council for Graduate Medical Education procedural requirements for liver biopsies, endoscopies, and paracenteses, which differs compared with pediatric kidney fellowships.

A short-lived accredited American Society of Transplantation Pediatric Renal Transplant Fellowship Training Program was introduced a few years ago, but this program was retired in 2009 for several reasons, including concerns that lengthening training to 4 or 5 years to accommodate transplant-specific training was not feasible for most trainees.

Although there is no specific requirement by the American Board of Pediatrics regarding how many biopsies must be performed (on native or transplant kidneys) during pediatric nephrology fellowship training, the results of this survey suggest that few programs have specific biopsy requirements for fellows. In addition, significant proportions of trainees had not participated in kidney transplant candidate evaluations or taken care of recent post-transplant patients in the inpatient setting or the outpatient setting for short-term and long-term longitudinal care. Arguably, nearly all pediatric nephrologists will care for kidney transplant recipients in their practice. Understanding and participation in the processes and organizations responsible for developing allocation policies for deceased donor organs, such as involvement in UNOS meetings, allow pediatric physicians to advocate for inclusion, diversity, and equity in the distribution of organs to our most vulnerable patients with organ failure. Ideally, a minimum number of required or recommended transplant experiences during training would be beneficial.

Our study has several limitations. We were unable to assess how many individual training programs were captured. Approximately 20–30 trainees graduate from pediatric nephrology fellowships each year. We attained over 50 individual responses from current fellows (still in training) suggesting good representation and 95 total responses from all comers. In addition, to minimize response burden, we limited our survey to 14 questions, and thus, many other questions remain. The survey did not capture deceased donor kidney transplant observations. We did not assess any training in ethics or diversity and inclusion, which are pivotal to ensuring equitable transplant access and candidate evaluations and which we would emphasize as an area to be included in training curriculum.

Our results suggest that trainees are graduating with mixed levels of competence in managing pediatric kidney transplants. Although some variation is to be expected, minimum standards and expectations across training programs would assure a more consistent exposure to portions of training that are considered to be core. This survey has uncovered an unmet need to standardize transplant-specific education for pediatric nephrology fellows.

## Future Directions

As a result of the findings of this survey, the Transplant Interest Group under the ASPN has worked to create a transplant curriculum. This is available through the ASPN website and is listed as “Transplant Training Core Curriculum.” We plan to re-evaluate the perceived competency of fellows after this has been available and used for several years.

## Supplementary Material

SUPPLEMENTARY MATERIAL

## Supplemental Material

Supplemental Appendix 1. Survey questions.
