# Supplementary material for: Assessment of Pediatric Kidney Transplant Experience and Exposure During Pediatric Nephrology Fellowship Training
Source: Kidney360. 2023 Jun 8;4(8):1139–42. doi: 10.34067/KID.0000000000000177 (PMC10476670; doi:10.34067/KID.0000000000000177)
Supplement: SUPPLEMENTARY MATERIAL [file kidney360-4-1139-s001.pdf]

Appendix 1

**Questions from the survey**
1. What year of training/post-fellowship are you currently?<br>a. 1st year fellow<br>b. 2nd year fellow<br>c. 3rd year fellow<br>d. Post 3rd year fellow, but still in training (4th or 5th year fellow)<br>e. 1st year post-fellowship junior faculty<br>f. 2nd year post-fellowship junior faculty<br>g. 3rd year post-fellowship junior faculty<br>h. Greater than 3 years post-fellowship faculty
2. Did/does your training institution perform routine surveillance biopsies on transplanted kidneys?<br>a. Yes<br>b. No, only biopsy if patient has a specific complication
3. How did/does your training institution perform biopsies on transplant kidneys?<br>a. Nephrology does the biopsy, no assistance from ultrasound<br>b. Nephrology does the biopsy, ultrasound technician assists<br>c. Interventional Radiology does the biopsy<br>d. Other, please explain
4. How many transplant kidney biopsies did you perform (in IR or with a nephrologist) during your training?<br>a. 1-10<br>b. 11-20<br>c. 21-30<br>d. 31-40<br>e. 41-50<br>f. Greater than 50<br>g. N/A
5. How many living kidney donation operations did you observe?<br>a. 1-3<br>b. 4-6<br>c. 7-9<br>d. 10-12<br>e. Greater than 12<br>f. N/A
6. How many deceased donation kidney harvests did you observe?<br>a. 1-3<br>b. 4-6<br>c. 7-9<br>d. 10-12<br>e. Greater than 12<br>f. N/A
7. How many kidney transplants from a living donor did you observe?<br>a. 1-3

b. 4-6<br>c. 7-9<br>d. 10-12<br>e. Greater than 12<br>f. N/A
8. How many kidney procurements from a deceased donor did you observe?<br>a. 1-3<br>b. 4-6<br>c. 7-9<br>d. 10-12<br>e. Greater than 12<br>f. N/A
9. How many fresh transplants did you care for directly as the primary team member (respond to calls, make medical decisions, etc.)?<br>a. 1-3<br>b. 4-6<br>c. 7-9<br>d. 10-12<br>e. Greater than 12<br>f. N/A
10. How many newly transplanted kidney recipients did you primarily care for at least 6 months from time of transplant?<br>a. 1-3<br>b. 4-6<br>c. 7-9<br>d. 10-12<br>e. Greater than 12<br>f. N/A
11. How many transplanted kidney recipients (not necessarily newly transplanted) did you follow for at least 6 months?<br>a. 1-10<br>b. 11-20<br>c. 21-30<br>d. 31-40<br>e. 41-50<br>f. Greater than 50<br>g. N/A
12. How many evaluations for potential kidney recipients were you directly involved in (including participation in selection committee meetings)?<br>a. 1-5<br>b. 6-10<br>c. 11-15<br>d. 16-20<br>e. 21-25<br>f. Greater than 25<br>g. N/A

13. Did your training program have a number required for any of these procedures?
    a. No
    b. Yes, provide further explanation.

14. How many UNOS meetings or regional committee meetings have you attended or listened in on?
    a. 0
    b. 1-2
    c. 3-4
    d. >4
